# Mucosal polyposis syndrome mimicking a rectal tumour

**DOI:** 10.1111/ans.70101

**Published:** 2025-03-25

**Authors:** Wei Ming Ong, Dhaval Joshi, Suat Chin Ng

**Affiliations:** ^1^ Department of Colorectal Surgery, Eastern Health Monash University Melbourne Victoria Australia; ^2^ Department of Anatomical Pathology Melbourne Pathology Melbourne Victoria Australia

19‐year‐old male presented with a 2.5‐year history of per rectal bleeding associated with defecation on the background of chronic constipation.

Per rectal examination revealed a moderately gaping anus with a small degree of full‐thickness rectal prolapse on bearing down and a villous‐like lesion palpated above the anorectal junction. An urgent colonoscopy showed multiple carpet‐like lesions extending from the dentate line to the rectosigmoid junction (Fig. 1). Initial biopsies identified these as inflammatory polyps, with no evidence of adenomatous or malignant changes, and no features suggestive of an underlying inflammatory bowel disease.

**Fig. 1:**
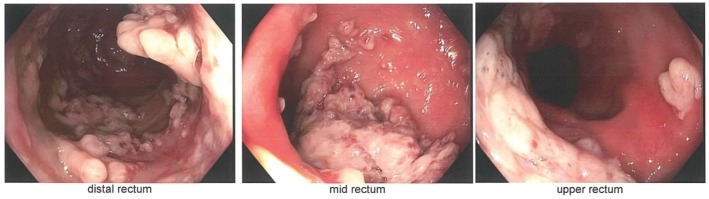
Endoscopic view of rectum.

A repeat endoscopy with large biopsies of the lesion was taken once more due to concern of a more sinister pathology. Histology demonstrated an ulcerated polypoid inflamed granulation tissue with features of mucosal prolapse (Fig. 2). He subsequently underwent a laparoscopic ventral rectopexy to correct the prolapse.

**Fig. 2:**
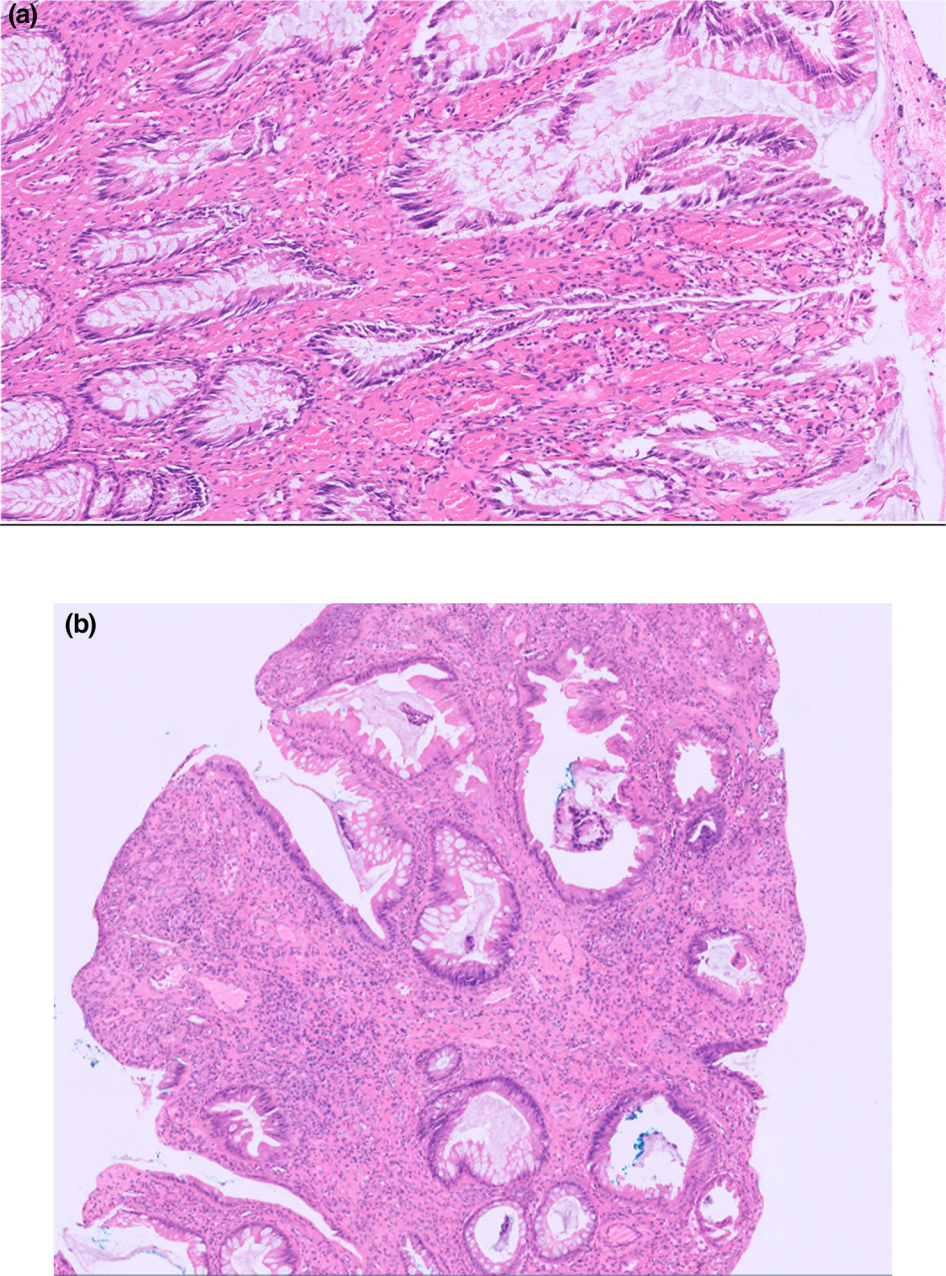
Histological features of mucosal polyp associated with rectal prolapse. (a) Features of mucosal prolapse with thickened muscularis mucosa with extension into lamina propria as well as vertically oriented smooth muscle fibres (arrows). (b) Ulcerated polyp with mucosal hyperplastic changes.

Mucosal prolapse syndrome (MPS) is an umbrella term for a broad range of conditions that stem from prolapsing mucosa.^1^ The most common entity of MPS is solitary rectal ulcer syndrome (SRUS) but other less‐known entities include proctitis cystica profunda (PCP) and inflammatory cloacogenic polyp.^2^ The incidence of MPS is estimated to be 1 in 100 000 and predominantly affects young adults.^1^, ^3^ The theorized pathogenesis of SRUS is due to excessive straining during bowel evacuation, leading to increased intra‐rectal pressures. The anterior wall of the rectal mucosa forcibly prolapses through the anal canal, which is paradoxically closed due to contraction of the puborectalis muscle. Recurrent trauma to rectal mucosa leads to inflammation and oedema, with the subsequent onset of ischaemia and regeneration resulting in the formation of either ulceration or mucosal polyposis.^4^

Clinical features of MPS include bleeding, tenesmus, constipation, passive mucus discharge, and anal itching. Endoscopic appearances of MPS vary from a shallow ulcerating lesion on a bed of erythematous mucosa to multiple ulcerative lesions or polypoid lesions.^5^ These polyps can mimic haemorrhoids, solitary rectal ulcer, villous adenoma, inflammatory bowel disease, or anorectal carcinoma. The macroscopic appearance of SRUS can be classified into ulcerative (55%), polypoid (24%) and flat (21%).^6^ In our patient, he had multiple polypoidal lesions throughout the lower rectum that appeared malignant at first glance.

Careful histopathological examination of the polyps is important in the diagnosis of mucosal prolapse syndrome due to its varied histological appearance and its ability to mimic other pathological lesions. Common features of MPS include glandular cystic dilation, ulceration, glandular architectural aberrations, thickening and disruption of the muscularis mucosae, and muscularisation of the lamina propria, resulting in characteristic fibromuscular obliteration of the lamina propria with disorientation of muscle fibres.^7^ The lamina propria may be oedematous and often shows increased numbers of fibroblasts.^8^

Treatment of MPS requires a multi‐disciplinary effort and may involve a surgical approach to correcting mucosal prolapse.

MPS is a mimicry for various pathological lesions. A meticulous history and examination, paired with a thorough endoscopic and histological examination of the biopsy specimens, are necessary to achieve a diagnosis.

## Author contributions

**Wei Ming Ong:** writer, methodology, editing. **Dhaval Joshi:** editing. **Suat Chin Ng:** conceptualisation, supervision, editing.
